# Supplementary material for: Quality and costs of commissioned vs. researcher-led NIHR research: retrospective cohort study of randomised controlled trials
Source: PLoS One. 2025 Apr 9;20(4):e0321425. doi: 10.1371/journal.pone.0321425 (PMC11981151; doi:10.1371/journal.pone.0321425)
Supplement: Attachment — Submitted filename: Response to Reviewers.docx [file pone.0321425.s002.docx]

|  |  |  |  | |
| --- | --- | --- | --- | --- |
|  |  | | | |
|  |  | | **centre for ACADEMIC PRIMARY CARE**  NIHR School of Primary Care Research  Bristol Medical School  Population Health Sciences  Canynge Hall, 39 Whatley Road  Bristol, BS8 2PS | |
|  |  |  | Ashley Hammond  Research Fellow  ashley.hammond@bristol.ac.uk  12^th^ February 2025 | |
| Dear Sonia, | | | |  |

**Re: Quality and costs of commissioned vs. researcher-led NIHR research: retrospective cohort study of randomised controlled trials (PONE-D-24-39387)**

Many thanks for your 3^rd^ January 2025 email inviting us to revise our manuscript. As requested, two versions are submitted (‘Revised Manuscript with Track Changes’ and ‘Manuscript’).

The table below describes our responses to both journal and reviewer comments, and the changes made to the manuscript where necessary. Please also note that after consideration we have revised our data availability statement in our resubmission and detailed below our reasoning for this.

Yours sincerely,

Ashley Hammond

# PONE-D-24-39387 Quality and costs of commissioned vs. researcher-led NIHR research: retrospective cohort study of randomised controlled trials

| **Comments** | **Author responses** | **Changes to manuscript** |
| --- | --- | --- |
| **Journal Comments** | |  |
| In your review, please pay careful attention to each comment made by Reviewers 1 and 2, providing detailed information on the screening of the database, and making the strategies for inter-rater reliability clearer. In the section Strengths and Weaknesses,you state that "[t]he main weaknesses [please correct typo] of this study is that there is no single accepted measure of research quality". You explain that you used two compensatory strategies and then provided Altmetric scores and relative citation ratio to avoid limiting your analysis to the JIF. You then report that the "findings were consistent across all selected quality measures". Using these compensatory strategies to manage the inherent subjectivity in quality assessment seems to be framed as negative. However, you used these strategies to enhance the overall assessment of quality. Please, clarify. | Thank you for this helpful insight. We agree that some clarification on this tone of this paragraph in our Discussion section would be useful, and so have added in some additional text to reaffirm that our use of the different measures of research quality enhanced the overall assessment. | Additional text added to lines 158-159, and 172-174. |
| Please ensure that your manuscript meets PLOS ONE's style requirements, including those for file naming. | We can confirm that the manuscript now meets all of PLoS One’s style requirements, including file names. | Updated title/author layout, headings, reference style and acknowledgements section. |
| Thank you for uploading your study's underlying data set. Unfortunately, the repository you have noted in your Data Availability statement does not qualify as an acceptable data repository according to PLOS's standards.  At this time, please upload the minimal data set necessary to replicate your study's findings to a stable, public repository (such as figshare or Dryad) and provide us with the relevant URLs, DOIs, or accession numbers that may be used to access these data. For a list of recommended repositories and additional information on PLOS standards for data deposition, please see [https://journals.plos.org/plosone/s/recommended-repositories](https://track.editorialmanager.com/CL0/https:%2F%2Fjournals.plos.org%2Fplosone%2Fs%2Frecommended-repositories/1/010f01942b9b2b48-eb6e1f1e-cd47-4207-ad23-3a8f12048500-000000/akx9W5uITJgGTwOpMdNLfzown5t5nBphFJS6GE4B9_k=191). | We apologies that the repository we noted was not considered acceptable. We have now uploaded the raw dataset used to conduct our view onto figshare as suggested, DOI is 10.6084/m9.figshare.28303580. | We have updated the data availability section of our submission form with the figshare URL for our dataset. |
| We note you have included a table to which you do not refer in the text of your manuscript. Please ensure that you refer to Table 3 in your text; if accepted, production will need this reference to link the reader to the Table. | We apologies for this error. We have now added references which link the reader to Table 3 in the main text. | Additional text added on lines 130 and 132. |
| **Reviewer 1 Comments** | |  |
| The link with supplementary data did not open. | We are not sure which link this reviewer is referring to, as we did not include any supplementary information. We have double-checked all links included in the paper and can confirm they all work correctly and link to the appropriate webpage. | No change, but double-checked and confirmed links included in lines 47/48, 71 and 79 are working correctly. |
| Maybe CP projects were about certain topic as neglected diseases, or diseases that are not prone to commercial interests but that are important as a public health issue. This should be included at Discussion topic. For example, in 2021, covid studies were by far more cited than others in health area. | We agree with the reviewer that this is an important point to address – that there are likely key differences in the research topics of commissioned compared to researcher-led projects. We have added in some additional discussion around this. | Additional text added to lines 164-168. |
| In the discussion, it was mentioned that there are no established criteria to measure the quality of research. However, another topic that could be addressed in the debate is the implementation of public policies and the increase in population coverage to address a specific health demand; these could also be used as quality indicators. I understand that conducting these analyses was not the objective of the study, but a paragraph reflecting on this topic should be included in the Discussion. | Thank you for this insight. We believe the addition of “Future quality assessments could include other more direct impact measures such as implementation of public policies and increasing the population health demand coverage being addressed” further strengthens our paper | Additional text added to lines 173-174. |
| Considering the importance of the funds invested in promoting health research in the United Kingdom, this work is extremely relevant to the discussion on the efficient use of financial resources, given that demand will always exceed the population's health needs. In a context of scarce resources, addressing this topic is highly significant. | We appreciate and thank the reviewer for their support with regards to the importance of this paper in setting a foundation for discussing the most efficient use of research funding. | No change. |
| **Reviewer 2 comments** | |  |
| Abstract - the Results section stated that there were some differences in the parameters measured, but the P value i not significant (there is one that is 0.05, but that should not be considered significant). The authors do not make such statements in the manuscript body. | We thank the reviewer for their comment. We report all p-values in the abstract and in the results section (Table 2 and 3). Whilst some were not significant, typically defined as p-value>0.05, two others we considered to be significant according to this standard definition of statistical significance (p-value ≤0.05), though we agree that the p-value of 0.05 is marginal. In light of this, we have amended the text in our abstract, discussion and conclusions section to reflect only those we considered statistically significant. | Removed text on lines 37-38, 141, and 183-184. |
| Introduction: More information should be provided for RPLs and CPs in terms of the length of the proposal, maximum amount of the grant, number of researchers? | Details on the length of each project proposal or the number of researchers on each project could not be collected due to lack of availability. Table 3 provides mean costs per participant and the total amount awarded for CPs and RLPs | No change. |
| Methods: The screening of the database and extractions of data - was it performed independently by the two authors? Was there any attempt to assess the agreement between them? | As this was not a systematic review, but a retrospective cohort study, the database screening was not performed independently by two authors. | No change. |
| Methods: The monographs should be described in more detail. Are they articles like the published articles? | We agree with the reviewer that an explanation of the monographs would be helpful, and have added extra text into the methods to make this clearer. | Additional text added to lines 83-85. |
| Methods: The calculation of RCR is not clearly explained. How was the area of research determined for individual projects? How many were such areas? | We explain in our methods section on lines 94-102 the definition of a RCR and how the RCR is calculated, with references for each. We agree that additional detail here explaining how the area of research is determined would be helpful, and so have added in some additional text. | Additional text added to lines 98-99. |
| Methods: Did the authors check the normality of data distribution, considering that the sample sizes were not big? | Normality of the data was checked, and a student’s t-test was considered to be appropriate for the data. To make this clear, we have added some additional text to our methods section. | Additional text added to lines 101, and 103-104. |
| Results, table 1: Did all projects had a monograph and a paper published? What is the difference? Also, what is a non-NIHR journal? NIHR seems to publish monographs and the difference is not well explained. | Data are provided in Table 1 around the number of papers for both CP and RLPs which had a monograph published, and a paper published in a peer-reviewed journal. We provide a link to The NIHR Journals Library website on line 79 which explains that the NIHR are the world’s first funder to produce six open-access journals publishing monographs of their funded projects. We agree this could be clearer in the methods, and so have added in some additional text to clarify. | Additional text added to lines 82-85. |
| Results, table 2: It is not clear when the papers were published to make sense of the impact factor data. | Journal impact factor data was collected in August 2024, irrespective of the publication date. Unfortunately, it was not possible to collect the journal impact factor at the time of publication for each paper. We have added in some additional text to our methods section to clarify this. | Additional text added to lines 84-86. |
| Results, table 2: The statistical test should be indicated in the table (Student t-test). | We thank the reviewer for this suggestion, and agree. We have added in the statistical test used into the headings of Table 2 and Table 3. | Additional text added to line 130, and 136. |
| Results, table 2: The authors state there were differences in parameters, but only the difference between journal impact factors was significant. | We thank the reviewer for their comment, however we consider the findings in Table 2 related to the Altmetric score with a p-value of 0.05 to be statistically significant, according to the standard definition of statistical significance (p-value ≤0.05). However, we do agree this could be considered marginal, and so we have added some additional text to our results section to make this clear. | Additional text added to lines 126-127. |
| Results, table 3: The authors state that "The mean award amount in CP was only marginally higher" but the p value was 0.59. | We agree with the reviewer that our wording used is not clear. We have amended the text in our results section to clarify. | Additional text added to lines 132-133. |
| Discussion: The main conclusions of the study should be based only on results with clear statistical significance. | We have amended the text in our discussion and conclusions section to reflect the statistically significant results. | Text amended on lines 149, and 191-192. |
